# Crisis Support VR: Bridging the Gap in Psychological First Aid Training -from initial idea to real virtual training

**DOI:** 10.1192/j.eurpsy.2025.214

**Published:** 2025-08-26

**Authors:** C. Hveem

**Affiliations:** Department of Preparedness and Security Protection, Sahlgrenska University Hosptial, Gothenburg, Sweden

## Abstract

**Abstract:**

This presentation will explore the development and implementation of the Crisis Support VR project, funded by the VGR Innovation Fund and involving five hospitals and primary care. In today’s society, the need for crisis support is more pressing than ever. However, we identified a significant gap in opportunities to practice Psychological First Aid. To address this, Region Västra Götaland collaborated with VirtualSpeech to create a system that has been tested by hospital staff, emergency services, and crisis support teams. This presentation will detail the project’s journey from its inception to its current state, highlighting the challenges and successes encountered along the way.

**Disclosure of Interest:**

None Declared

